# Interface Structure of MoO_3_ on Organic Semiconductors

**DOI:** 10.1038/srep21109

**Published:** 2016-02-16

**Authors:** Robin T. White, Emmanuel S. Thibau, Zheng-Hong Lu

**Affiliations:** 1 Department of Materials Science and Engineering, University of Toronto, 184 College St., Toronto, ON, M5S 3E4, Canada; 2 Department of Physics, Yunnan University, Kunming, China

## Abstract

We have systematically studied interface structure formed by vapor-phase deposition of typical transition metal oxide MoO_3_ on organic semiconductors. Eight organic hole transport materials have been used in this study. Ultraviolet photoelectron spectroscopy and X-ray photoelectron spectroscopy are used to measure the evolution of the physical, chemical and electronic structure of the interfaces at various stages of MoO_3_ deposition on these organic semiconductor surfaces. For the interface physical structure, it is found that MoO_3_ diffuses into the underlying organic layer, exhibiting a trend of increasing diffusion with decreasing molecular molar mass. For the interface chemical structure, new carbon and molybdenum core-level states are observed, as a result of interfacial electron transfer from organic semiconductor to MoO_3_. For the interface electronic structure, energy level alignment is observed in agreement with the universal energy level alignment rule of molecules on metal oxides, despite deposition order inversion.

Transition metal oxides (TMOs) are often used in organic electronic devices due to their excellent hole injection characteristics, by acting as a p-dopant^1^,^2^,^3^. TMOs such as MoO_3_^4^, V_2_O_5_^5^, WO_3_^6^, and many others have also been shown to reduce the energy offset between electrode and organic materials by acting as a surface modification layer, or electrode buffer layer. In device applications, these TMOs are involved in charge exchange between substrate and adsorbed organic molecules, resulting in favorable energy level alignment and improved performance.

In an inverted OLED structure^7^,^8^,^9^,^10^, inverted organic photovoltaic cells (OPVs)^11^,^12^,^13^,^14^, and organic field-effect transistors (OFETs)^15^ the TMO is deposited on top of the organic semiconductor. For OLEDs in display applications with n-channel field-effect transistors, it is necessary to have the cathode in contact with the driving element of the circuit^7^, thus requiring an inverted top emission device in which the structure is flipped so that the top anode is transparent and the bottom cathode is reflective. Wide band gap molecules such as 4,4′ -N,N′ -dicarbazole-biphenyl (CBP), which has a deep ionization potential of 6.0 eV, are known to form charge transfer complexes with very high electron affinity TMOs such as MoO_3_. These TMOs have been found to be very good p-type dopants for hole transport materials (HTMs)^1^,^2^,^3^. The use of such chemical doping has been found to improve the performance of many optoelectronic devices such as OLEDs, by reducing interfacial charge injection barriers, thus increasing carrier injection^4^,^16^. Previous reports have also shown that doping CBP with MoO_3_ improved optoelectronic device performance by a factor of up to five orders of magnitude with 22 mol%^17^. Utilizing the inverted deposition method has been shown to have similar effects to co-deposition due to the diffusion of MoO_3_ in the organic semiconductor, improving device performance^10^. However, compared to the non-inverted case, very little research has gone into the effects of deposition order inversion on the resulting interface between TMO and organic semiconductor.

Ultraviolet photoelectron spectroscopy (UPS) has been used previously to study the electronic structure of the interface formed by deposition of MoO_3_ on top of polymer semiconductor F8BT^2^. MoO_3_ was found to diffuse into the F8BT film and act as a *p*-dopant and a new interface state close to the Fermi level was also observed. The new state and band bending were proposed as the cause for the improved hole-injection from MoO_3_. In this paper, we report the first photoelectron spectroscopy study of vapour-deposited prototypical TMO MoO_3_ on various archetypal organic HTMs, whose various HOMO and LUMO energy levels can be found in Fig. 1. We study the resulting physical, chemical and electronic aspects of the interface and highlight differences compared to the non-inverted deposition order. It is shown that deposition order inversion shows important changes in these interfacial properties, which can have a substantial impact on device performance.

## Experimental Procedures

Photoemission spectra were collected with a PHI 5500 Multi-Technique system, using monochromated Al Kα radiation (hν = 1486.7 eV) for XPS core-shell measurements and a non-monochromated He Iα photon source (hν = 21.22 eV) for UPS. Work function measurements were measured at a take-off angle of 88°. During UPS (valence and work function), the sample was held at a bias of −15 V relative to the spectrometer.

Organic molecules were thermally evaporated from an alumina crucible using an *in situ* transfer arm evaporator described elsewhere^18^, at a rate of 0.3 Å/s, as measured by a calibrated quartz crystal microbalance (QCM), to a thickness of approximately 15 nm, using typical small organic molecular film density. This thickness was chosen such that the underlying substrate, highly ordered pyrolitic graphite (HOPG), had little effect on the organic semiconductor layer, while thin enough to prevent charging during measurement.

All MoO_3_ films were grown by vacuum sublimation from 99.9% pure MoO_3_ powder placed into a 10cc alumina crucible in a Knudsen cell. MoO_3_ was evaporated at a temperature between 500–550 °C to obtain a deposition rate of 0.1–0.2 Å/sec. The evaporation source was positioned 31 cm away from the sample, at an angle of 35° relative to sample normal^19^. The chamber pressure during deposition was approximately 2 × 10^−9^ torr.

**Figure 1:**
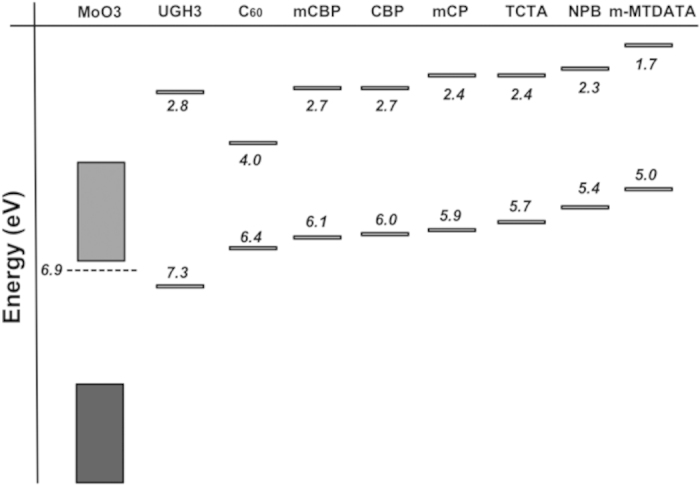
Energy levels as measured by UPS, with band gap and LUMO positions taken from literature^1^,^17^,^18^,^19^,^29^,^30^,^31^,^32^.

Supporting Table S1 lists the eight organic molecules and their chemical structures used in the experiment. Figure 1 shows the various frontier energy-level positions of the materials used in this work, as measured by UPS, unless otherwise stated.

## Results and Discussion

### Interface Physical Structure

A unique result of this study is the observation of MoO_3_ diffusion as a result of deposition order inversion. In a non-inverted deposition (organic on MoO_3_), substantial diffusion at this interface does not occur because a higher density and stable metal or metal oxide is the underlying substrate. When an inverted deposition order is employed, the high temperature and heavy MoO_3_ clusters have enough kinetic energy to penetrate into the underlying organic layer and diffuse through the typically low density, amorphous structure of these films.

**Figure 2:**
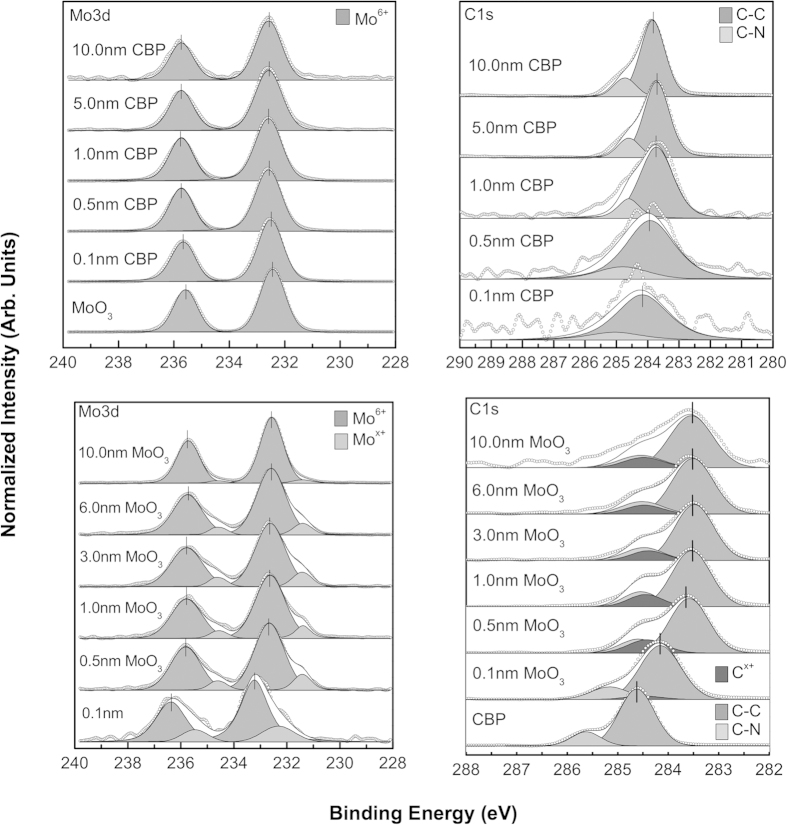
XPS high-resolution core-level scans of C1*s* and Mo3*d* peaks for CBP on MoO_3_ system (top row) and the MoO_3_ on CBP inverted deposition system (bottom row). Shift in core level peaks indicate band bending as well as new peak formation from perturbation of electron density by interaction with MoO_3_.

Based on the inelastic mean free path (IMFP) of photoelectrons in an MoO_3_ film, attenuation of the C1*s* core-shell peak should occur after only a few nanometers of MoO_3_ overlayer deposition. However, as shown in Fig. 2, even after 10 nm, there remains a strong C1*s* signal. One possible explanation for this delayed peak attenuation is the formation of clusters or islands of MoO_3_. To investigate this, we analyze the intensity ratio of C1*s* to Mo3*d* core-level peaks for incrementally deposited MoO_3_ on the organics, found in supporting Figure S1. The exponential decay of the signal intensities for all organic molecules indicates that it there is in fact layer-by-layer growth of the MoO_3_ film rather than island formation^20^. Therefore, the reason for the variation in exponential decay is varying degrees of penetration of the MoO_3_ into the organic film, which effectively decreases the overlayer thickness of MoO_3_ by some scale factor, *g*. The *g* values for the various organic/MoO_3_ systems effectively relates the amount of MoO_3_ diffusion they exhibit. Therefore, employing the well-known model of a thin overlayer on top of a thick substrate^21^, and using *g* as a fitting parameter, we have


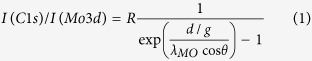


where *λ*_MO_ is the IMFP of the MoO_3_ overlayer, *d* is the overlayer thickness, *θ* is the photoemission take off angle (75° in this case) and *R* is the ratio of standards for the materials used, 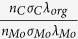. Here, *n* is the photoelectron emitter concentration and σ is the particular emitter’s cross section for photoemission. The IMFPs of the various materials used in this study can be found in supporting Table S2.

**Figure 3:**
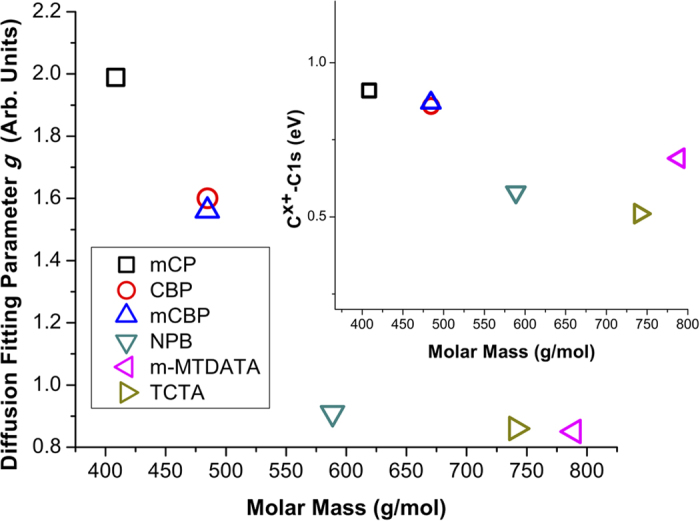
Relationship between molar mass of organic molecules and the fitting parameter *g*, indicating relative MoO_3_ diffusion, obtained using equation 1. *g* values less than unity can be accounted for by imperfect thickness monitor calibration. Inset shows similar trend for emergent carbon peak relative to C-C peak of the C1*s* envelope.

Previously in the literature, the diffusion of MoO_3_ has been suggested to be a result of its high sublimation temperature and heavy mass clusters that will have sufficient kinetic energy to penetrate into the underlying organic material due to low glass transition temperature^2^,^10^. This model is consistent with the fact that in the non-inverted deposition order, no diffusion is observed, seeing as the organic materials have substantially lower sublimation temperatures than MoO_3_. It has been suggested by Zhao *et al.*^10^ that the amount of MoO_3_ diffusion is dependent on the thermal stability of the organic molecule, i.e., its sublimation temperature. When comparing the molecules used in this study, mCP, CBP and mCBP showed the most diffusion, with more massive molecules NPB, TCTA and MTDATA displaying the least. Figure 3 shows a clear trend of increasing diffusion with decreasing organic molar mass, which also supports the aforementioned diffusion by high kinetic energy model. Seeing as the thermal stability of organic molecules exhibiting similar weak intermolecular forces is largely dictated by their molecular mass, this also agrees with previous observation, though it may prove a more practical metric for assessing an organic molecule’s diffusibility.

### Interface Chemical Structure

The penetration of TMOs such as MoO_3_ into the organic layers enhances hole-transport characteristics, acting as a *p-*dopant by forming charge transfer states^1^,^2^. To date, the chemical and electronic interaction between MoO_3_ and organic molecules used in the inverted device structure has not been properly characterized, leaving many questions related to degradation mechanisms, device performance and the development of new materials.

**Figure 4:**
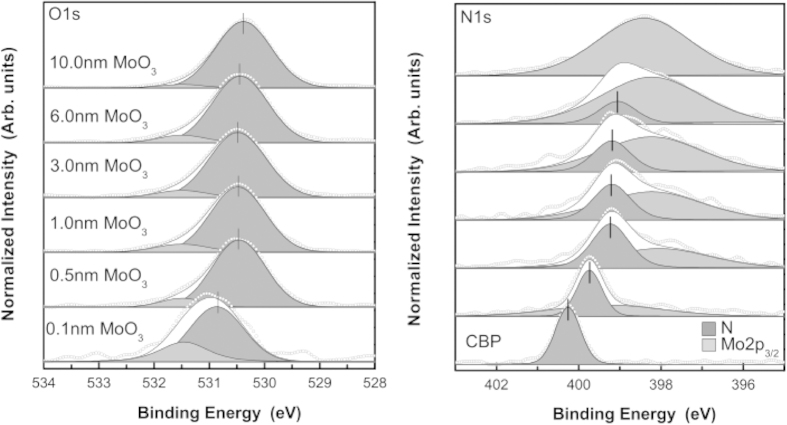
XPS high-resolution spectra of O1*s* and N1*s* core-level peaks for MoO_3_ on CBP. The Mo2*p*_3/2_ peak overlaps with the N1*s* peak.

Unlike in the non-inverted deposition order, when analyzing the interface in the inverted case, there is initially a strong carbon photoemission signal, which when paired with molybdenum’s high photoemission cross section, allows for detailed interactions to be observed. Figure 2 compares the C1*s* and Mo3*d* core-level peaks for both deposition orders of CBP and MoO_3_. The two peaks initially present in the C1*s* spectra represent, the C-N and C-C bonding states, from highest to lowest binding energy, respectively. The open circles are raw experimental data, the greyscale-filled peaks are the curve-fitted, de-convolved peaks and the solid line shows their sum, i.e., the fitted elemental envelope. For MoO_3_ on CBP, peak shifts attributed to band bending are seen due to charge transfer and doping by diffusion of MoO_3_ into the underlying CBP layer. The shift in the Fermi level causes the C1*s* envelope to move to lower binding energy after donating charge to MoO_3_ and the Mo3*d* to shift to higher binding energy after receiving charge. These shifts are supported by the O1*s* and N1*s* spectra shown in Fig. 4, in which similar band bending shifts are seen. Evidence of a charge transfer state is found in the emergence of additional core-shell peaks, denoted C^x+^ and Mo^x+^, also shown in Fig. 3. While the formation of reduced Mo^5+^ by interaction with organic substrate has been previously observed^22^, the counter-part C^x+^ state has not been previously reported, most likely due to the diffusion at the interface, stronger interaction and the higher C1*s* signal intensity resolved in the inverted deposition system. As shown in the inset of Fig. 3, the energetic separation between the new C^x+^ peak and the lowest binding energy peak of the C1*s* envelope (C-C bond state), is found to display a significant overall decrease for the heavier molecules (NPB, TCTA, *m-*MTDATA) compared to the much lighter ones (mCP, CBP, mCBP). When coupled with the interfacial diffusion data, this suggests that increased MoO_3_ diffusion and intermixing may result in a larger energetic separation in the emergent carbon charge transfer state, relative to the C-C bond state. Furthermore, the appearance of a second interfacial O1*s* state (leftmost O1*s* peak in Fig. 4) suggests that oxygen also plays an active role in the complex interaction between MoO_3_ deposited on organic molecular films. The experimental results are consistent for all organic molecules used in this report (see Table 1 and supporting Figure S2). The variation in N1*s*/C1*s* intensity ratio before and after 0.5 nm MoO_3_ deposition, summarized in Table 1, is within the assumed curve fitting error of up to 20%, arising especially due to Mo2*p*_3/2_ convolution with the N1*s* peak, and is thus deemed insignificant. This rules out the possibility of molecular breakdown followed by mass loss as a cause for new peak formation. Therein are also tabulated the observed binding energy separations between charge transfer states (C^x+^, Mo^x+^) and the C1*s* (C-C) and Mo3*d*_*5/2*_peaks.

### Interface Electronic Structure

As mentioned previously, the electronic structure at the organic/MoO_3_ interface plays a very important role in the performance of many organic electronic devices. The injection barrier for charge carriers in a given device architecture must be minimized, which will result in decreased contact resistance^23^.

MoO_3_ was deposited on eight different organic semiconductors and their work functions and valence states were observed using UPS. The incremental layer-by-layer deposition/analysis method was used to observe the change from organic valence states to occupied MoO_3_ states. Since the kinetic energy of UV emitted electrons is low, the escape depth from within the sample is also very small. For this reason, UPS is a very surface sensitive technique.

**Figure 5:**
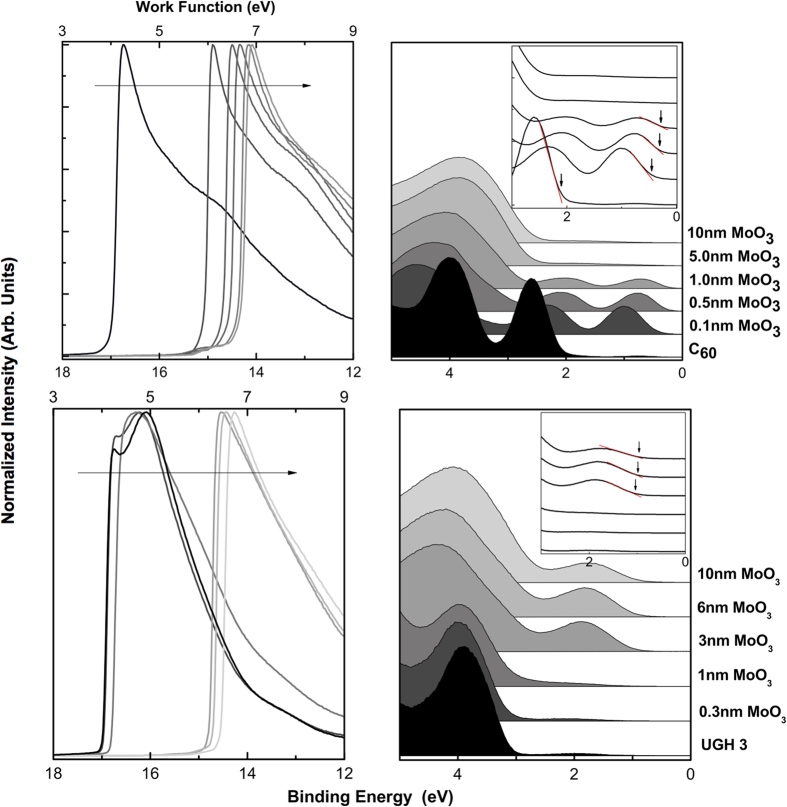
UPS spectra showing shifting secondary electron cut-off, indicating surface interface dipole change and valence spectra showing Fermi level pinning with HOMO for C_60_ (top) and no pinning for UGH 3 (bottom). Inset shows the shifting position of the HOMO peak. The Fermi level is calibrated to 0 eV.

**Figure 6:**
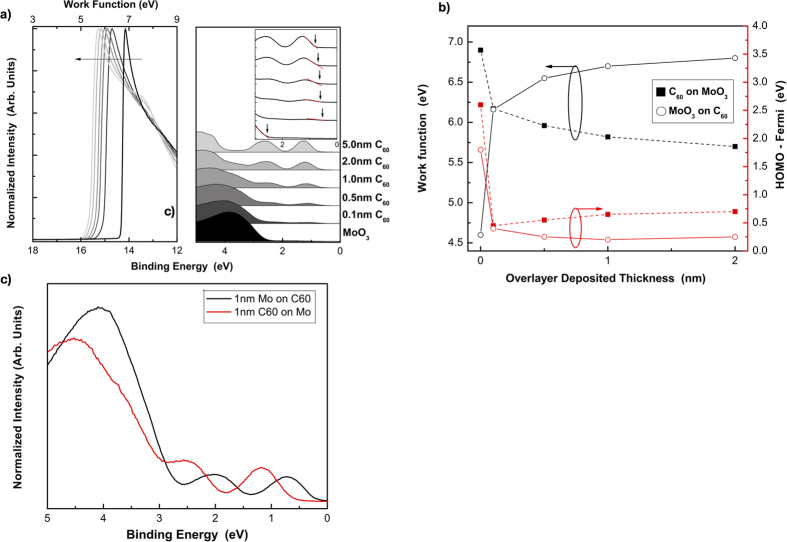
(**a**) UPS spectra of C_60_ on MoO_3_. (**b**) Summary of work function and HOMO offset for both C_60_ on MoO_3_ (filled in squares) and MoO_3_ on C_60_ (open circles) as a function of deposited overlayer thickness. The lines are a guide to the eye. (**c**) Valence spectra with 1nm of deposited material.

The increased band bending in the inverted deposition order has already been shown via the XPS core level shifts, shown in Fig. 3, and further UPS of C_60_ and MoO_3_ corroborates these results. As can be seen in Fig. 5, as MoO_3_ is deposited on C_60_, the C_60_ valence states shift to lower binding energy before they attenuate, as does the secondary electron cutoff (shown on the left), indicating vacuum level shift congruent with band bending. Figure 6 further exemplifies the asymmetric nature of this interface based on deposition order, showing that in the non-inverted order, the p-doping is less prominent, and a larger HOMO-Fermi is measured at the interface. In the non-inverted incremental deposition of C_60_ on MoO_3_ (Fig. 6a), less shifting of the HOMO toward the Fermi level is detected, as compared to the inverted case. Figure 6b further shows that MoO_3_ incrementally deposited on C_60_ displays a larger change in work function (vacuum level shift), as well as a smaller HOMO-Fermi, compared to C_60_ on MoO_3_. The associated raw interfacial UPS valence band data for both deposition orders of C_60_ and MoO_3_ is displayed in Fig. 6c, again showing a smaller HOMO-Fermi in the inverted case. This reduction in the HOMO-Fermi could be a result of the increased interaction and *p*-doping of the film from penetration of MoO_3_ into the underlying organic layer. The formation of defect states and reduced MoO_3−x_ species could possibly pin the HOMO closer to the Fermi level, as various changes in overlayer film properties have been discussed by the calculation of HOMO offset by Ley *et al.*^24^.

**Figure 7:**
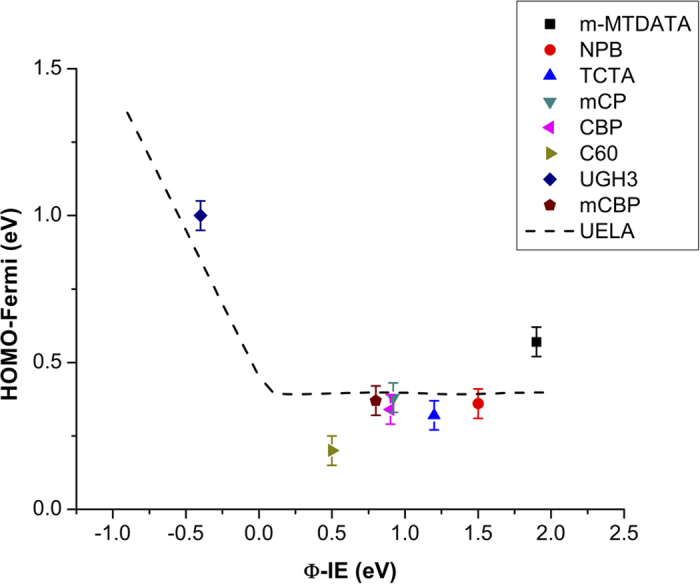
Energy level alignment data and theory^26^, (dashed line) showing the HOMO-Fermi as a function of the difference between the organic’s ionization energy (IE) and MoO_3_ work function (Φ).

Figure 5, top, shows an example of Fermi level pinning for MoO_3_ on C_60_, as is seen here in all cases where charge transfer is favorable, i.e., when the organic’s ionization energy is smaller than the MoO_3_ work function. When this is not the case, Fermi level pinning does not occur, as shown for MoO_3_ on UGH3 in Fig. 5, bottom. This agrees with the Universal Energy Level Alignment (UELA) rule of molecules on metal oxides^25^, which is the dashed line plotted in Fig. 7, calculated according to Chai *et al.*^26^. All data in Fig. 7 were taken from the 1nm UPS spectra of MoO_3_ on organic, found in supporting Figure S3.

When two solids are far away from one another, their vacuum levels can be considered to be in alignment. As they come into contact with one another, charge transfer is allowed to occur which forms a dipole layer at the interface, changing the vacuum level position and aligning the Fermi levels of both materials. This charge transfer can be a result of interfacial chemical reactions, redistribution of electron cloud or any other type of charge rearrangement^27^. Upon charge transfer, the corresponding electric field locally shifts the HOMO, LUMO and vacuum levels by the same amount. The induced dipole, as a result of charge transfer, is sufficient enough to pin the HOMO to the Fermi level minimizing the energy barrier between electrode and organic layer.

Besides this interfacial dipole model, there is also the existence of interface states, especially in MoO_3_. It has been shown by Greiner *et al.*^25^. that a reduced MoO_3−x_ has defect states that lie very close to the Fermi level, which could become occupied upon charge transfer of electrons to MoO_3−x_. From the penetration of MoO_3_ into the underlying organic material and subsequent electron transfer to MoO_3_, the organic semiconductor is effectively *p*-doped. When the concentration of charge carriers is increased, the Fermi level moves towards the associated transport level, namely the HOMO.

## Conclusion

**Figure 8:**
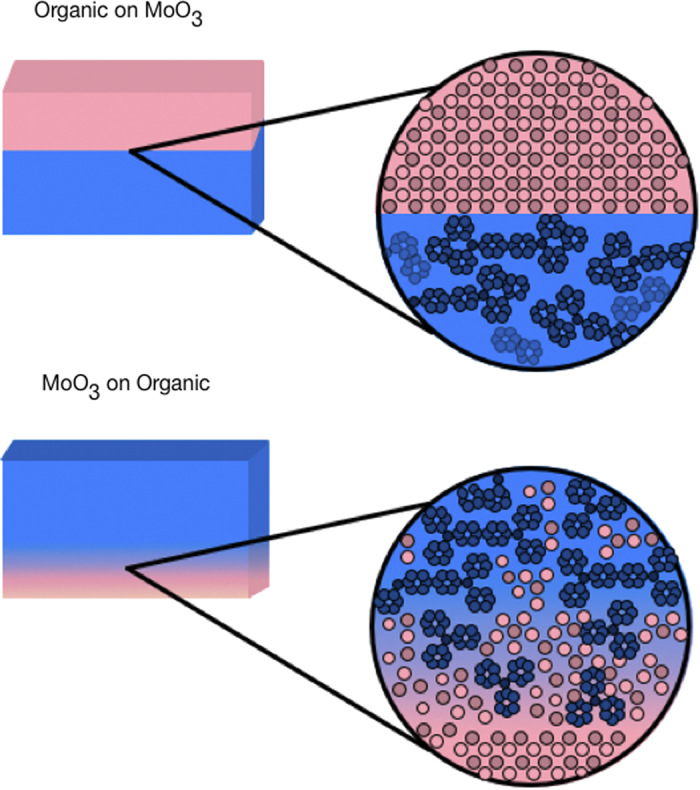
Schematic diagrams illustrating the effect of deposition order on physical interface formation. When deposited on the organic films, MoO_3_ diffuses into the underlying layer.

Using photoelectron spectroscopy (XPS and UPS), an understanding of the interfacial interaction between several vapour-phase-deposited organic materials and MoO_3_ has been established. It was found that the high kinetic energy MoO_3_ clusters were able to penetrate into the underlying soft organic material. The relative amount of diffusion was observed based on a simple over-layer model with a fitting parameter related to the overlayer thickness. Plotting this relative diffusion fitting parameter against molar mass of the molecules revealed a clear trend, with the heavier and larger molecules displaying less MoO_3_ diffusion, i.e., a more discrete boundary. An illustration of such diffuse and discrete interfaces is shown in Fig. 8.

In the inverted deposition order, shifts in high-resolution XPS core level peaks indicate band bending and increased charge transfer between MoO_3_ and the organic semiconductors. This was shown to be unobservable when conducting the experiment in the non-inverted manner, as the photoelectron cross-section for carbon is small and the interface boundary would be discrete, so by the time there is sufficient C1*s* signal, the interface is no longer within the probing depth of XPS. The formation of new C1*s* and Mo3*d* peaks from donating and receiving charge, respectively, was also shown for the first time, confirming the occurrence of significant charge transfer.

Increased band bending and charge transfer in the inverted deposition order were further confirmed by UPS measurements, which showed the pinning of HOMO energy levels closer to the Fermi level, when thermodynamically favorable. This inverted deposition order is consistent with the UELA rule of organic semiconductor molecules on metal oxides^28^. Minimization of the HOMO-Fermi level is vitally important for device applications, as this decreases contact resistance, providing efficient charge injection into the device.

## Additional Information

**How to cite this article**: White, R. T. *et al.* Interface Structure of MoO_3_ on Organic Semiconductors. *Sci. Rep.* **6**, 21109; doi: 10.1038/srep21109 (2016).

## Supplementary Material

Supplementary Information

## Figures and Tables

**Table 1:** N1*s*/C1*s* intensity ratios before and after 0.5 nm MoO_3_ deposition and binding energy of new C^x+^ and Mo^x+^ peaks, relative to the C1*s* (C-C bond, lowest C1*s* binding energy state) and Mo3*d*_5/2_ peaks, respectively.

Molecule	N1*s*/C1*s* (0 nm MoO_3_)	N1*s*/C1*s*(0.5 nm MoO_3_)	C^x+^-C1*s*Chemical Shift [eV]	Mo^x+^- Mo3*d*_5/2_Chemical shift [eV]
mCP	0.11	0.10	0.91	−1.11
CBP	0.10	0.08	0.86	−1.25
mCBP	0.10	0.09	0.87	−1.08
NPB	0.09	0.10	0.58	−1.07
m-MTDATA	0.12	0.09	0.69	−1.08
TCTA	0.13	0.11	0.51	−1.06
C_60_	n/a	n/a	0.59	−1.24
