# Supplementary material for: Interface Structure of MoO3 on Organic Semiconductors
Source: Sci Rep. 2016 Feb 16;6:21109. doi: 10.1038/srep21109 (PMC4754744; doi:10.1038/srep21109)
Supplement: Supplementary Information [file srep21109-s1.doc]

Supplementary Information

Interface Structure of MoO_3_ on Organic Semiconductors

Robin T. White, Emmanuel S. Thibau* and Zheng-Hong Lu

**Figure S1**. Experimental results and fitted curves for MoO_3_ incrementally deposited on 6 organic molecules. Equation 1 was used to fit the experimental data and extract values for the relative diffusion fitting parameter, *g*.

Figure S2. C1*s* and Mo3*d* XPS high-resolution core level peaks for mCBP, mCP, NPB, TCTA and MTDATA which show similar interaction with MoO_3_ forming charge transfer species. Shift in core level peaks indicate band bending as well as new peak formation from perturbation of electron density by interaction with MoO_3_.

Figure S3. UPS spectra of organic molecules used in this study. Each figure is labeled to show thickness of MoO_3_ deposited on each pristine organic film. Inset shows the position of the HOMO onset.

Table S1. Structure and name of the organic compounds used in this work.

| **Molecular name** | **Abbreviation** | **Structure ^[1-4]^** |
| --- | --- | --- |
| *N*,*N*′-Bis(naphthalen-1-yl)-*N*,*N*′-bis(phenyl)benzidine | NPB | 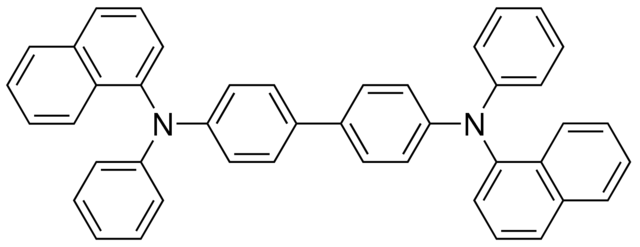 |
| 4,4′-Bis(*N*-carbazolyl) -1,1′-biphenyl | CBP | 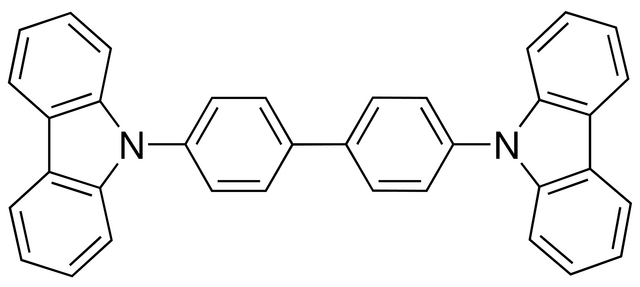 |
| Tris(4-carbazoyl-9-ylphenyl)amine | TCTA | 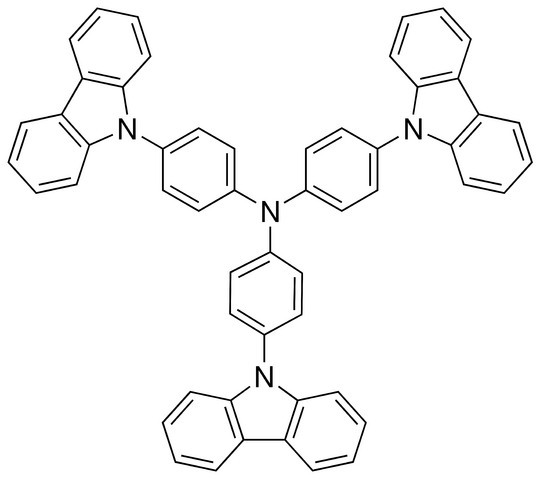 |
| 4,4′,4′′-Tris[phenyl(*m*-tolyl)amino]triphenylamine | m-MTDATA | 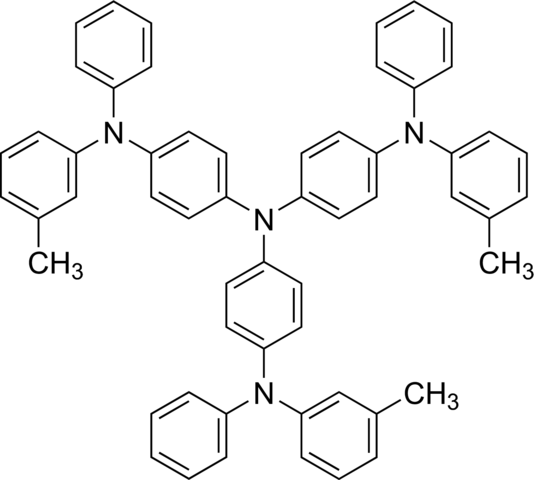 |
| 1,3-Bis(*N*-carbazolyl)benzene | mCP | 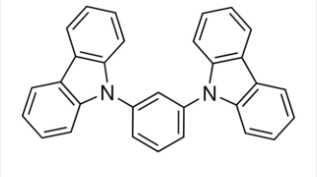 |
| 9-[3-(3-carbazol-9-ylphenyl)phenyl]carbazole | mCBP | 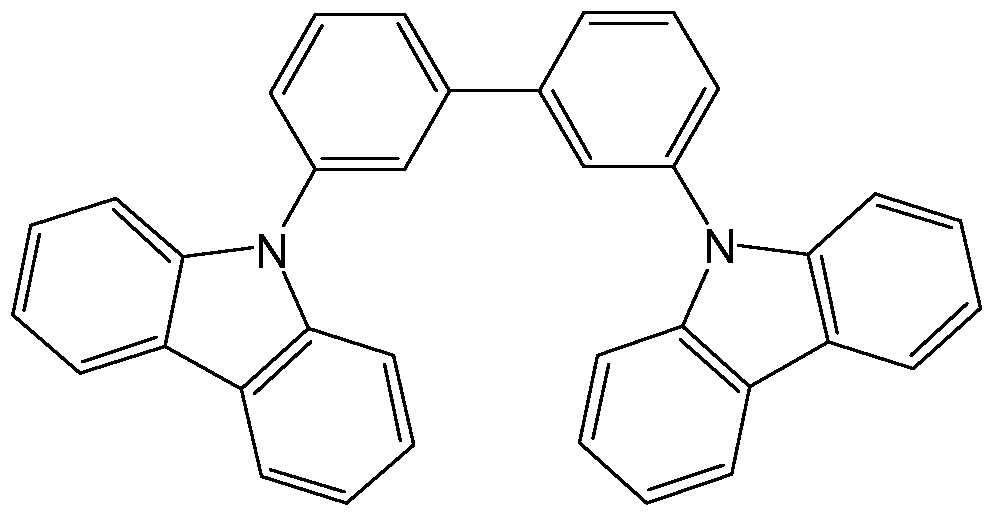 |
| 1,3-Bis(triphenylsilyl)benzene | UGH 3 | 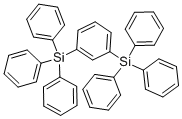 |
| Fullerene –C_60_ | C_60_ | 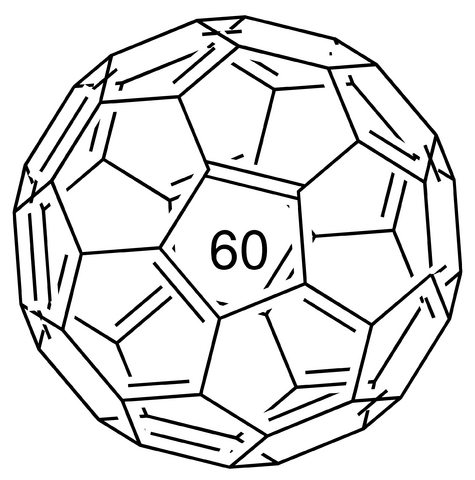 |

**Table S2**. Values used in the calculation of the inelastic mean free path.^[5]^

| **Molecule** | **Band gap (eV)** | **Valence electrons** | **Molar Mass (g/mol)** | **Inelastic mean free path (**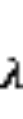**) (nm)** |
| --- | --- | --- | --- | --- |
| CBP | 3.3 | 178 | 484.59 | 3.285 |
| NPB | 3 | 218 | 588.74 | 3.296 |
| mCBP | 3.3 | 178 | 484.59 | 3.285 |
| TCTA | 3.2 | 272 | 740.89 | 3.297 |
| mCP | 3.7 | 50 | 408.49 | 3.427 |
| MTDATA | 3.2 | 296 | 789.02 | 3.326 |
| MoO_3_ | 2.8 | 19 | 143.94 | 2.4 |

**References**

[1] S. Yin, Z. Shuai, and Y. Wang, *Chem. Inf. Model.* **2003**, *43*, 3.

[2] S. Schmidbauer, A. Hohenleutner, and B. König, *Adv. Mater.* **2013**, *25*, 15.

[3] T.-L. Chiu and P.-Y. Lee, *IJMS*. **2012**, *13,* 12.

[4] J. Lee, *ETRI J,* **2009**, *31*, 6.

[5] C. J. Powell and A. Jablonski, NIST Electron Inelastic-Mean-Free-Path Database - Version 1.2, National Institute of Standards and Technology, Gaithersburg, MD (2010).
